# Gonadogenesis in the Bearded Dragon (*Pogona vitticeps*, Agamidae): A Comprehensive Histological Analysis from Gonadal Ridge Formation to Testicular and Ovarian Development

**DOI:** 10.3390/biology15120977

**Published:** 2026-06-22

**Authors:** Izabela Rams-Pociecha, Paulina C. Mizia, Rafal P. Piprek

**Affiliations:** 1 Department of Comparative Anatomy, Institute of Zoology and Biomedical Research, Faculty of Biology, Jagiellonian University, Gronostajowa 9, 30-387 Krakow, Poland; 2 Doctoral School of Exact and Natural Sciences, Jagiellonian University, 30-387 Krakow, Poland

**Keywords:** bearded dragon, gonad, gonadal development, lizard, ovary, sexual differentiation, testis

## Abstract

The bearded dragon (*Pogona vitticeps*) is the most commonly kept pet lizard and a promising model organism for studies of sex determination and gonadal development. Despite its potential, the morphological basis of gonadogenesis in this species remains poorly characterized. Here, we provide a comprehensive histological characterization of gonad development in *P. vitticeps* using serial paraffin and semi-thin sections, supplemented by morphometric analyses. Gonadal ridges first appeared at stage S28 as bilateral thickenings of the coelomic epithelium, coinciding with primordial germ cell colonization; by S28/29, a recognizable cortex and medulla were already present. The first morphological differences between male and female gonads appeared at S29/30. In differentiating testes, well-defined testis cords with a central lumen formed rapidly, while the cortex became thin and retained only scattered germ cells. Testicular development was characterized by rapid lumen formation within the testis cords, resulting in their early transformation into seminiferous tubules, followed by elongation and coiling of the tubules, maintenance of a stable tubule diameter, and a transient mitotic arrest of germ cells, with proliferation resuming at stage S36. In differentiating ovaries, the cortex remained thick and multilayered, with a progressive increase in germ cell number reflecting active oogonial proliferation. The ovarian medulla expanded substantially, and from S36 onward, lacunae developed within the medullary cords. No meiotic cells were observed at any examined stage. These results provide an essential morphological framework for future molecular and experimental studies of sex determination and gonadal differentiation in this species and in squamates more broadly.

## Simple Summary

The bearded dragon is one of the most popular pet lizards in the world and has great potential as a scientific model for studying how sex is determined during embryonic development. Before an embryo develops into a male or female, the reproductive organs—the gonads—pass through an early stage in which they look the same in both sexes. Only later do they differentiate into testes or ovaries. In this study, we tracked the step-by-step formation and development of the gonads in bearded dragon embryos using microscopic analysis of tissue sections. We found that the gonads first appear on embryonic day 8 and already begin to differ between males and females just one day later—an unusually rapid transition compared to other reptiles. In developing testes, characteristic tubular structures form quickly, while in ovaries, the outer layer remains thick and the number of germ cells increases progressively. The ovaries also develop unusually large internal cavities compared to other reptile species, which may be related to the bearded dragon’s ability to produce large clutches of up to 30 eggs. These findings provide an essential foundation for future research into how genes and incubation temperature together control sex determination in this species, including the remarkable phenomenon of temperature-induced sex reversal.

## 1. Introduction

Gonads are pivotal organs for reproduction, responsible for the production of gametes and the secretion of sex hormones. The differentiation process of gonads, transforming primordial structures into either testes or ovaries, is a complex interplay of genetic, molecular, and environmental factors. This intricate process determines an organism’s sex and involves precise morphogenetic changes, beginning with the formation of the gonadal ridges.

Gonadal ridges are paired gonadal primordia that, in all vertebrates, develop on the surface of the mesonephros bilaterally on either side of the dorsal mesentery [1]. They arise as a result of the proliferation of coelomic epithelial cells and project into the body cavity, representing the earliest morphological manifestation of gonadal formation.

Gonadal ridges, as aggregations of coelomic epithelial cells, serve as the target site for migrating primordial germ cells (PGCs) [2]. Within the gonadal primordia, these cells lose their capacity for active migration and are henceforth referred to as gonocytes or gonial cells [3]. For simplicity, all cells observed in the developing gonads throughout this study will be collectively referred to by the broadest term—germ cells. An additional cell population that joins the forming gonads consists of mesenchymal cells derived from the mesonephros. The growing gonadal ridges subsequently transform into undifferentiated gonads, composed of a primary cortex (also known as a germinal epithelium) and a primary medulla [1]. Sex cords grow from the primary cortex as elongated aggregates of somatic cells, frequently containing germ cells. As they extend toward the center of the gonad, these cords constitute the so-called medullary cords. In differentiating male gonads, they are referred to as testis cords. Initially, the germ cells are located in both gonadal regions. During the undifferentiated gonad stage, sex determination genes are expressed within gonadal cells. Following the period of sex determination, the first distinctions between male and female gonads become apparent, thus marking the onset of gonadal sex differentiation.

In differentiating testes, germ cells disappear from the cortex, which transforms into a thin, single-layered epithelium [4]. In the medulla, however, germ cells (prospermatogonia) become enclosed by differentiating epithelial cells (Sertoli cells) thereby forming testis cords, which will subsequently give rise to seminiferous tubules through the formation of a central lumen within the cords [5,6]. The testis cords and seminiferous tubules are surrounded by a basement membrane that separates them from the interstitium, in which Leydig cells differentiate and secrete testosterone. In differentiating ovaries, the reverse pattern is observed: germ cells (oogonia) are retained in the cortex, while those in the medulla undergo regression. Within the cortex, germ cells enter meiosis and ovarian follicles ultimately form. The ovarian medulla also expands and, in many species, gives rise to cords with a lumen that develops into the ovarian cavities (lacunae) [7]. Individual species, even within reptiles, differ with respect to, for example, the degree of differentiation of medullary cords and their lacunae in the ovary, as well as the developmental stage at which meiosis is initiated in the embryonic ovary.

Regarding gonadogenesis in reptiles, the majority of studies have been conducted on the American alligator, sea turtles, and the red-eared slider [5,8,9,10,11,12,13,14]. However, despite the extraordinary diversity of squamates, which comprise 12,374 species and account for 98.46% of all extant reptiles (as of September 2025), detailed histological studies of gonadogenesis remain limited to only a few species. Histological studies of gonadogenesis have been conducted on three gecko species (*Eublepharis macularius*, *Correlophus ciliatus*, *Lepidodactylus lugubris*), the veiled chameleon (*Chamaeleo calyptratus*), the skink *Niveoscincus ocellatus*, the anguid lizard *Barisia imbricata*, the agamid *Calotes versicolor*, and the phrynosomatid lizard *Sceloporus aeneus* [7,15,16,17,18,19,20].

Squamates exhibit remarkable diversity in morphology, enabling them to inhabit a wide range of environments, and display considerable variation in reproductive strategies and sex determination systems. They diverged from the remaining reptiles approximately 240 million years ago, during the Middle Triassic—a timespan sufficient for the evolution of numerous characteristics distinguishing them from crocodilians and turtles [19].

The bearded dragon (*Pogona vitticeps*) is the most popular lizard kept as a pet [21]. Due to the easy accessibility of eggs, straightforward breeding, and the possibility of obtaining large quantities of biological material, this species has the potential to become a model organism for developmental studies, including investigations into sex determination. Such investigations initially require a thorough and detailed characterization of the developmental processes underlying the formation of testicular and ovarian architecture. In *P. vitticeps*, the heterogametic sex is female (ZZ/ZW); however, temperature exerts a significant influence on sex determination. When eggs are incubated at elevated temperatures (above 32 °C), genetic males (ZZ) undergo sex reversal and develop as fertile females [22].

The aim of the present study was to provide a detailed characterization of the processes occurring in the developing gonads of the bearded dragon, from the emergence of the earliest gonadal primordia, through the indifferent gonad stage and sexual differentiation of the gonads, to the establishment of definitive testicular and ovarian architecture.

## 2. Materials and Methods

### 2.1. Animals

Eggs of the bearded dragon (*P. vitticeps* Ahl, 1926, Agamidae, Acrodonta, Iguania) were obtained from private breeders in Kraków (Lesser Poland Voivodeship, Poland) in 2019 and 2020. Eggs were collected on the day they were laid and incubated on a wet vermiculite mixture (200 g of vermiculite and 160 mL of water) at 28 °C. In earlier studies, a 50:50 sex ratio was obtained at this temperature [18]. The number of embryos tested at consecutive stages is presented in Supplementary Table S1. Embryos were staged according to the developmental tables of Wise et al. [23]. The diagnostic features used for stage identification are presented in Supplementary Table S2. This study was conducted in accordance with the Act on the Protection of Animals Used for Scientific or Educational Purposes (Dz.U. 2015, poz. 266).

### 2.2. Histological Analysis

Embryos dissected from eggs were fixed in Bouin’s solution overnight, dehydrated, and embedded in paraffin (Paraplast, Sigma, P3683, St. Louis, MO, USA). Samples were serially sectioned at 6 μm using a microtome (ThermoScientific, HM355S, Waltham, MA, USA). Slides were stained with Harris-modified hematoxylin and picroaniline according to the Dubreuil procedure [24,25]. For semi-thin sections, samples were fixed in Karnovsky’s solution, rinsed in cacodylate buffer and post-fixed in 1% osmium tetroxide solution [26]. The samples were then dehydrated and embedded in Epon 812. Sections were stained with a 1:1 mixture of methylene blue and Azure. Images were taken using a Nikon Eclipse E600 light microscope (Tokyo, Japan).

### 2.3. Statistics

Measurements were performed using Fiji software (version 2.9.0). Statistical analyses and graphical representations were conducted using GraphPad Prism 8.

## 3. Results

### 3.1. The Gonadal Ridges and Undifferentiated Gonads

**Figure 1:**
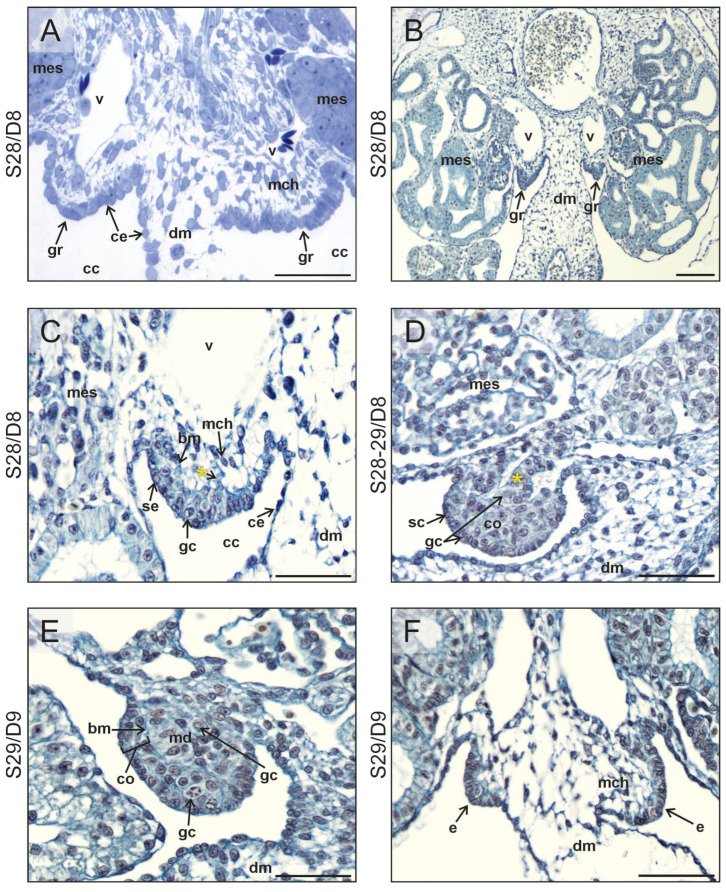
Gonadal ridges and undifferentiated gonads in *P. vitticeps*. (**A**) Gonadal ridges prior to PGC colonization at S28/D8. On a semi-thin section, the gonadal ridges are clearly visible as two thickenings of the coelomic epithelium (ce) protruding into the coelomic cavity (cc), situated on both sides of the dorsal mesentery (dm). The thickened coelomic epithelium of the gonadal ridges borders an accumulation of mesenchyme (mch). Dorsal to the gonadal ridges, the posterior cardinal veins (v) and mesonephroi (mes) are present. (**B**,**C**) Gonadal ridges colonized by PGCs at S28/D8. The gonadal ridge (gr) is covered by the superficial epithelium of the gonad (se), which is continuous with the coelomic epithelium. Germ cells (gc) are closely associated with the inner surface of the gonadal epithelium. The gonadal epithelium is multilayered, and some of its cells protrude into the interior of the gonad, which may signal the onset of medullary cord formation (yellow asterisk). The gonadal epithelium is separated from the mesenchymal region by a basement membrane (bm). (**D**) The undifferentiated gonad at S28–29/D8 is prominently protruded into the coelomic cavity; the superficial epithelium of the gonad is markedly thickened, forming the primitive cortex (co), from which medullary cords arise (yellow asterisk). Somatic cells (sc) and germ cells are visible within the cortex. (**E**) In the undifferentiated gonad at S29/D9, a distinct subdivision is apparent between the primitive cortex and the gonadal interior (primitive medulla), which is occupied by medullary cords. These structures are separated by basement membranes (bm). Germ cells are present in both the cortex and the medullary cords. (**F**) The posterior segment of the gonad (e, epigonad) at S29/D9 resembles the gonadal ridge in organization, with a relatively thin epithelium in which only sparse germ cells are embedded. Scale bars: (**A**,**C**–**F**)—50 μm; (**B**)—100 μm.

The earliest signs of gonadal ridge formation were observed at stage S28, corresponding to the 8th day of incubation (D8) (Figure 1). At this stage, transverse sections show gonadal ridges prior to colonization by primordial germ cells (Figure 1A). The ridges appear as a pair of thickened coelomic epithelium bulges located between the dorsal end of the dorsal mesentery and the medial surface of the mesonephroi (Figure 1A,B). The dorsal mesentery of the gut runs between the two gonadal ridges. A pair of posterior cardinal veins passes above the gonadal ridges. The mesonephroi are positioned lateral to the gonadal ridges. In two individuals examined at this developmental stage, the gonadal ridges had not yet been colonized by primordial germ cells and therefore consisted exclusively of somatic cells derived from the coelomic epithelium. These cells are tightly packed and adopt the morphology of simple cuboidal epithelium, locally transitioning to columnar epithelium (Figure 1A), whereas the coelomic epithelium outside the gonadal ridges forms a simple squamous epithelium. Mesenchymal cells occupy the space dorsal to the gonadal ridge epithelium (Figure 1A).

In two individuals at this stage (S28/D8), germ cells were already present within the gonadal ridges (Figure 1B,C). Thus, colonization of the gonadal ridges by primordial germ cells takes place at this stage. Germ cells are found within the thick, multilayered epithelium covering the gonad. These cells are easily distinguished from somatic cells by their round shape, larger size, and large, spherical, lightly stained nuclei (mean germ cell diameter: 9.6 μm). Somatic cells have irregular shapes, with thin processes extending between the germ cells, and possess small, oval, darkly stained nuclei. As the gonadal epithelium thickens, the gonadal ridges project further into the body cavity (Figure 1B,C). The mean epithelial thickness is 26.5 μm, compared with 14.7 μm in uncolonized epithelium. The thickened gonadal epithelium represents the primordium of the gonadal cortex. Within this early cortex, the epithelial cells fall into several distinct types: 1. flattened somatic cells located between and surrounding the germ cells, 2. cells positioned at the gonadal surface, separating the germ cells from the body cavity, 3. elongated columnar epithelial cells arranged in a palisade pattern, spanning from the gonadal surface to the basement membrane of the epithelium, 4. cells located adjacent to the basement membrane of the cortex, 5. cuboidal cells forming the lateral surfaces of the gonads.

The space between the gonadal epithelium and the wall of the posterior cardinal vein is occupied by loosely arranged mesenchymal cells embedded in extracellular matrix. The cortex is separated from the mesenchyme by a basement membrane, which is visible as a faintly blue-stained band of the extracellular matrix. Protrusions of the gonadal epithelium into the mesenchymal zone are present (marked with yellow asterisks in Figure 1C), possibly representing the onset of sex cord formation.

At stage S28/29 (D8), the gonadal ridges have already taken the form of undifferentiated gonads (Figure 1D). They project clearly into the body cavity. Their structure is visibly divided into a cortical and a medullary region. The cortical region is thicker compared to the cortex of the gonadal ridge at stage S28 (mean: 34.1 μm versus 26.5 μm). The sex cords (medullary cords) previously suggested by epithelial protrusions at stage S28 are now clearly present in the medullary region. The proximal ends of the medullary cords are connected to the cortex, while their distal ends terminate adjacent to the wall of the renal corpuscle (Figure 1D). Germ cells are found in the cortex as well as in the medullary cords (Figure 1D). The medullary cords are surrounded by a basement membrane that is continuous with the basement membrane lining the inner surface of the cortex.

At stage S29 (D9), a division into cortex and medulla is clearly visible (Figure 1E). The cortex surrounds the medulla and is demarcated by the basement membrane. The cortex takes the form of an epithelial layer covering the gonad, while the medulla consists of a cluster of medullary cords. The germ cells are present in both the cortical epithelium and the medullary cords (Figure 1E). The posterior segment of the gonad (epigonad) is underdeveloped, resembles a gonadal ridge, and contains only a cortex with isolated germ cells and mesenchyme (Figure 1F).

### 3.2. Sexual Differentiation of Gonads

**Figure 2:**
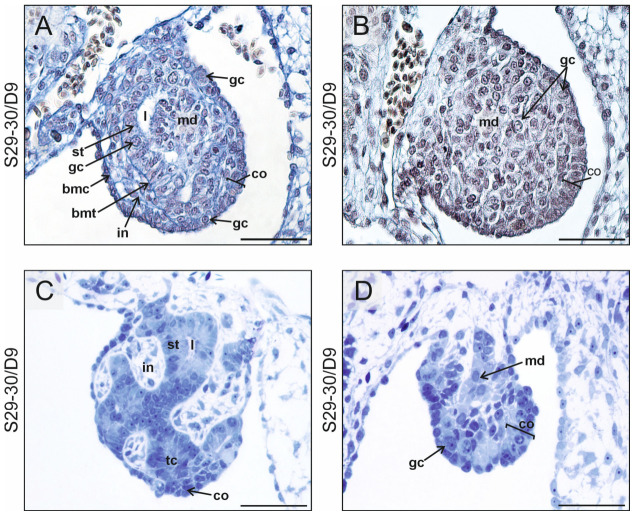
Sexual differentiation of gonads. (**A**,**C**) Differentiating testes are recognizable at S29–30/D9 by the presence of well-organized testis cords (tc). These cords contain germ cells (gc). At this stage, a lumen (l) begins to appear locally within the testis cords, indicating the onset of their transformation into seminiferous tubules (st). The testis cords and seminiferous tubules form the gonadal medulla (md). The primitive cortex is thin and contains few germ cells. Basement membranes are clearly visible, surrounding the testis cords and seminiferous tubules, and separating the primitive cortex (co) from the gonadal interior. Somatic cells, which give rise to the interstitium (in), accumulate between the basement membrane of the cortex (bmc) and the basement membrane surrounding the testis cords and seminiferous tubules (bmt). A semi-thin section (**C**) through the differentiating testis shows that the testis cords are anchored within the primitive cortex. Also clearly visible is the thin, flattened epithelium that remains from the primitive cortex following the loss of germ cells at the periphery of the testis. (**B**,**D**) Differentiating ovaries are recognizable at S29–30/D9 by a better-developed cortex, which takes the form of a multilayered epithelium containing numerous germ cells, while the center of the ovary is occupied by a medulla (md) formed by poorly demarcated cords. Scale bars: (**A**–**D**)—50 μm.

The first sexual differences in gonadal structure are observed at stage S29/30 (D9) (compare Figure 2A,C with Figure 2B,D). In differentiating testes, the medullary cords become readily distinguishable as well-differentiated, epithelialized structures. They are clearly separated from the surrounding mesenchyme (differentiating interstitium) by conspicuous basement membranes. Already during the early stages of testicular differentiation, rapid lumen formation occurs within the testis cords as a result of the progressive separation of the epithelial cord cells (differentiating Sertoli cells). Consequently, the testis cords undergo an early transformation into seminiferous tubules, which are already present in differentiating testes (Figure 2A,C). The cortex of the differentiating testis becomes thin, retaining only rare, single germ cells (typically 4–5 germ cells visible per transverse section) (Figure 2A,C). Semi-thin sections clearly demonstrate the transformation of the gonadal cortex into a thin, single-layered epithelium continuous with the testis cords and seminiferous tubules (Figure 2C).

Differentiating ovaries exhibit a structure similar to that of undifferentiated gonads (Figure 2B,D). The cortex of the differentiating ovaries remains thick, forming a multilayered epithelium that contains a higher number of germ cells than in testes (mean: 6–7 germ cells per transverse section, compared with 2–3 in the testes). The mean cortical thickness in differentiating testes is 6.3 ± 1.4 μm, whereas in differentiating ovaries it is 17.6 ± 6.3 μm. The central region of the ovary is occupied by medullary cords that lack a lumen, exhibit no clear epithelial differentiation, and lack distinct basement membranes, in contrast to testis cords (Figure 2D).

### 3.3. Testis Development

**Figure 3:**
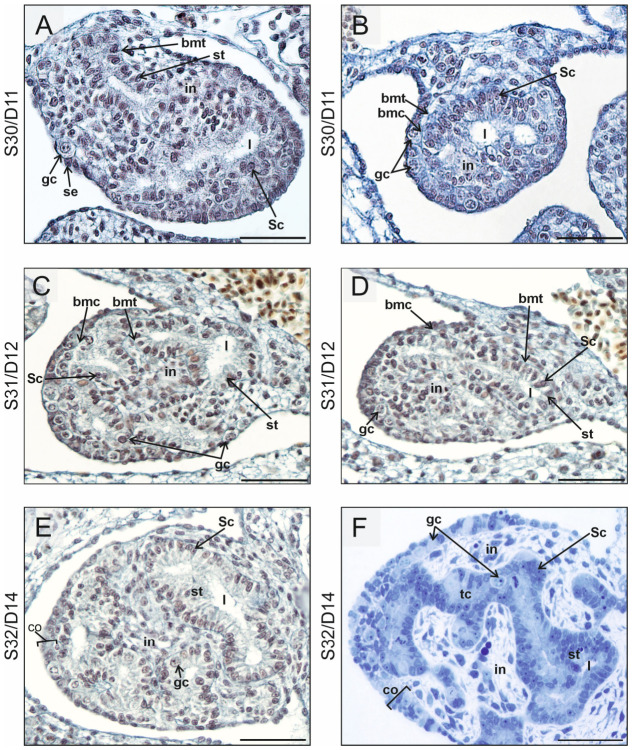
Developing testes at S30/D11 (**A**,**B**), S31/D12 (**C**,**D**) and S32/D14 (**E**,**F**). At each of these stages, seminiferous tubules (st) are visible within the testis; their walls are composed of a single layer of Sertoli cells (Sc), and a lumen (l) is present in the center of the tubules. The surface of the gonad is formed by a thin cortex (co), consisting of the superficial epithelium (se) of the gonad and germ cells (gc). Germ cells are also present within the walls of the seminiferous tubules (st). The cortex is separated from the gonadal interior by a poorly defined cortical basement membrane (bmc). The testis cords are separated from the interstitium (in) by well-developed basement membranes of the seminiferous tubules (bmt). (**F**) Semi-thin sections provide a clear illustration of the structural differences between the gonadal epithelia (cortex and testis cords (tc) and seminiferous tubules) and the interstitium, which is composed of scattered cells. Scale bars: (**A**–**F**)—50 μm.

In subsequent developmental stages, the testes display a similar organizational pattern to that observed during their differentiation at stage S29/30 (D9). The majority of the testis cords are already differentiated into seminiferous tubules, as evidenced by the presence of a distinct lumen within their central region. The testis cords and seminiferous tubules contain germ cells enclosed by Sertoli cells (Figure 2A,C and Figure 3). The testes are covered by a thin epithelium, which in certain regions also contains germ cells. Both the superficial epithelium and the testis cords/seminiferous tubules are separated from the interstitium by basement membranes. The testis cords are anchored in the surface epithelium of the cortex (Figure 2C).

**Figure 4:**
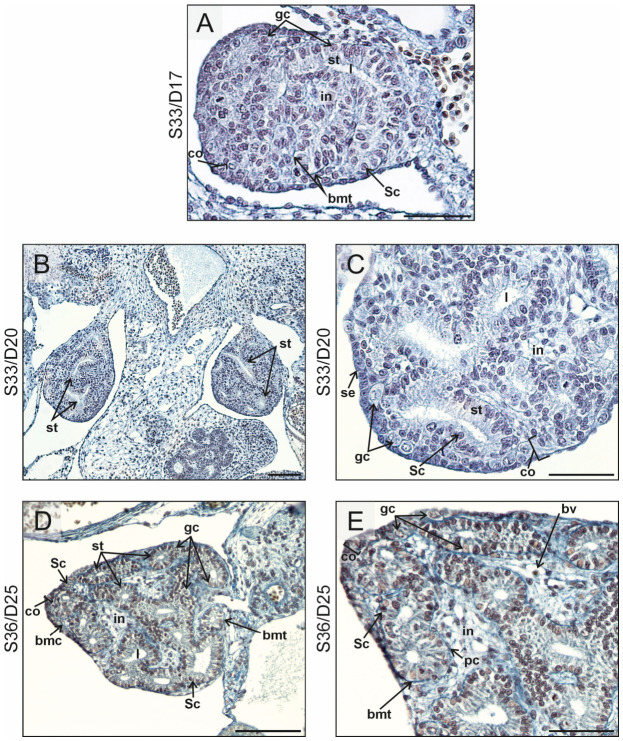
Developing testes at S33/D17 (**A**), S33/D20 (**B**,**C**), and S36/D25 (**D**,**E**). At these stages, the number of seminiferous tubules (st) is increased compared with earlier stages. The cortex (co) is reduced to a thin superficial epithelium (se) containing only scattered germ cells (gc) and is separated from the gonadal interior by a cortical basement membrane (bmc). The seminiferous tubules (st) contain Sertoli cells (Sc) and germ cells and are enclosed by distinct basement membranes (bmt). At S36/D25, blood vessels (bv) are evident within the interstitium (in), together with cells bearing crescent-shaped nuclei that are closely associated with the basement membranes of the seminiferous tubules (st). These cells are presumed to represent differentiating peritubular cells (pc). Scale bars: (**A**,**D**)—100 μm; (**B**,**C**,**E**)—50 μm.

**Figure 5:**
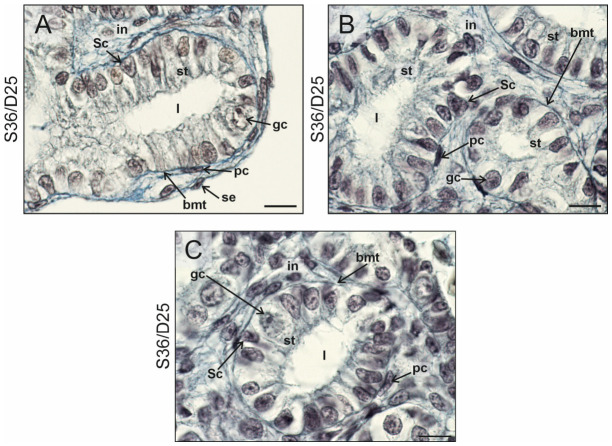
Higher magnification of the testis structure at S36/D25 (**A**–**C**). The wall of the seminiferous tubules (st) is clearly visible, formed by a single layer of Sertoli cells (Sc) lying on the basement membrane of the seminiferous tubules (st) (bmt). Germ cells (gc) are also present within the cord wall. A lumen (l) is present inside the seminiferous tubules, and the interstitium (in) is located between the cords. Peritubular cells (pc) with crescent-shaped nuclei are arranged around the seminiferous tubules (st). The gonad is covered by the superficial epithelium (se). Scale bars: (**A**–**C**)—10 μm.

**Figure 6:**
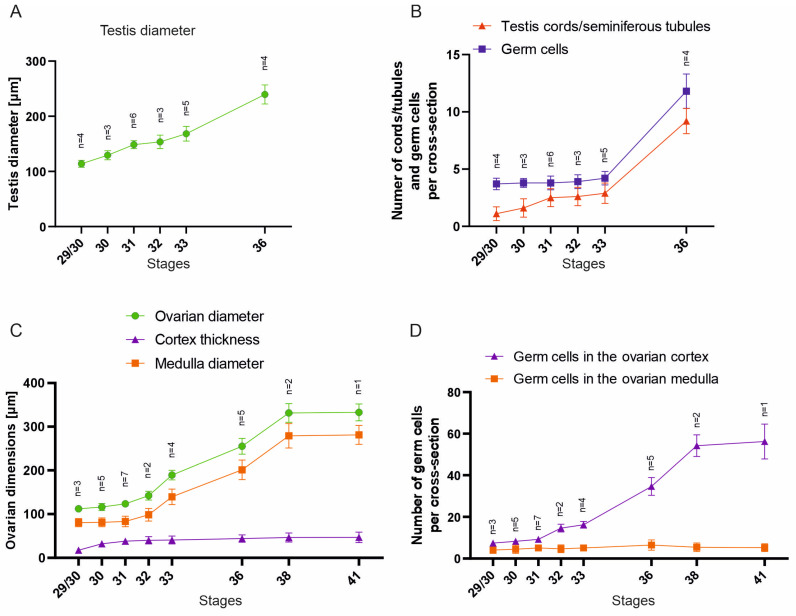
Changes in morphometric parameters of developing testes and ovaries in *P. vitticeps*. (**A**) Changes in the mean diameter of the testes at individual developmental stages. (**B**) Changes in the mean number of testis cords or seminiferous tubules and the mean number of germ cells observed in a single transverse section of the testis. (**C**) Changes in the mean ovarian diameter, cortical thickness, and medullary diameter. (**D**) Changes in the mean number of germ cells observed in the cortex and medulla in a single ovarian section. All values represent means of measurements performed in the widest central region of the gonads.

During embryonic development, the testes grow considerably (Figure 3, Figure 4 and Figure 5). The increase in gonad diameter is presented in Figure 6A and Supplementary Table S3. As the gonad grows, the number of testis cords/seminiferous tubules visible in transverse section increases (Figure 6B; Supplementary Table S4). From stage S33/D17 onwards, only seminiferous tubules were observed in the testes, whereas testis cords were no longer present (Figure 4). The increasing number of seminiferous tubules visible in transverse section may not reflect the formation of new cords, but rather their elongation and coiling. Elongation of the seminiferous tubules is observed across all examined stages as they become coiled (Figure 4B). The diameter of the seminiferous tubules does not change throughout the examined stages, measuring 39.8 ± 7.6 μm. The wall of the seminiferous tubules takes the form of a simple columnar or pseudostratified epithelium (when the nuclei of Sertoli cells are positioned at different heights), resting on a basement membrane. The mean diameter of Sertoli cell nuclei is 8.3 ± 0.4 μm. The somatic cells within the tubules, differentiating into Sertoli cells, have oval nuclei that are typically basally positioned (e.g., Figure 3B, Figure 4C and Figure 5). Germ cells within the seminiferous tubules are located basally and are rarely visible in earlier developmental stages (mean: 2–4 germ cells per transverse section through the middle segment of the gonad) (e.g., Figure 3F and Figure 5). The number of germ cells within the seminiferous tubules is slightly higher compared to those present in undifferentiated gonads. In the early stages of testicular development, the number of germ cells within the seminiferous tubules does not increase, indicating mitotic arrest (Figure 6B; Supplementary Table S5).

Numerous germ cells are more easily visible in the thinned cortex of the testes (e.g., Figure 3B,C and Figure 4C). Across all examined stages, the testicular cortex takes the form of a thin epithelium with locally occurring germ cells. Where germ cells are present, the epithelium is thicker (e.g., Figure 3F). The number of cortical germ cells visible per transverse section remains constant and does not change across successive stages, amounting to approximately 5–7 germ cells. This constant number of germ cells has been maintained since the undifferentiated gonad stage, suggesting that cortical germ cells neither proliferate nor undergo apoptosis during testicular development in the examined stages.

The most advanced developmental stage for which testicular samples were available in this study was S36 (D25) (Figure 4D,E and Figure 5). At this stage, the histological structure of the seminiferous tubules is well organized and differentiation of interstitial cells is observed. The number of germ cells within the seminiferous tubules is markedly higher compared to previous stages, indicating the onset of cell division (Figure 6B; Supplementary Table S5). The arrangement of cells within the seminiferous tubules is more regular compared to previous stages (Figure 5). The nuclei of Sertoli cells are positioned at a uniform basal level. The nuclei of neighboring Sertoli cells are spaced apart, suggesting that these cells are no longer tightly packed. The lumen within the tubules is wide compared to previous stages. As in previous stages, germ cells (prospermatogonia) rest on the basement membrane of the tubules. In addition to germ cells present within the seminiferous tubules, a proportion of germ cells is found in the surface epithelium of the testis. Cortical germ cells are concentrated only at the site most distant from the gonadal mesentery (Figure 4D). At this location, the epithelium covering the testis takes the form of a multilayered epithelium. The remainder of the gonadal surface is covered by a simple squamous epithelium (Figure 4D and Figure 5A). From S36 (D25) onwards, blood vessels are present in the interstitial tissue, surrounded by interstitial cells (likely including Leydig cells) (Figure 4E). From this stage onwards, cells with oval, flattened nuclei, which are likely differentiating peritubular cells, are found adjacent to the outer surface of the seminiferous tubules (Figure 4E and Figure 5B). In earlier stages, no blood vessels were observed within the testes, and the interstitial cells formed a uniform mass filling the spaces between the seminiferous tubules (compare Figure 4C,E).

### 3.4. Ovary Development

**Figure 7:**
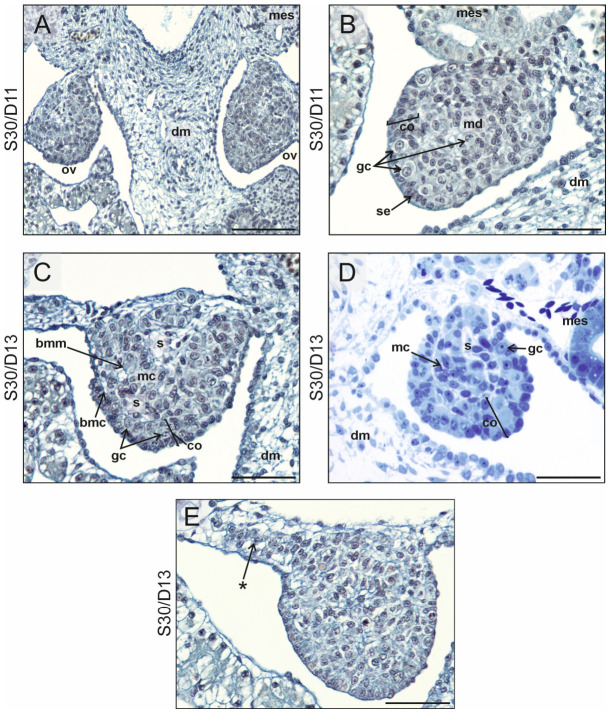
Developing ovaries. (**A**,**B**) Developing ovaries (ov) at S30/D11 are situated on both sides of the dorsal mesentery (dm), ventral to the mesonephros (mes). The thick cortex (co) constitutes the peripheral zone of the organ and is composed of superficial epithelial cells (se) and germ cells (gc). Germ cells are also present in the medulla (md). (**C**–**E**) In developing ovaries at S30/D13, medullary cords (mc) are visible in the gonadal center. These cords are surrounded by the basement membrane of the medullary cords (bmm). The basement membrane of the cortex (bmc) is also visible. Between the two basement membranes lies the stroma (s), formed by scattered cells. (**D**) A semi-thin section clearly illustrates the structural distinction between the gonadal epithelia (cortex and medullary cords) and the stromal compartment. (**E**) The asterisk indicates a cord of somatic cells extending from the gonadal medulla toward the mesonephros. Scale bars: (**A**)—100 μm; (**B**–**E**)—50 μm.

Like the testes, the developing ovaries are located on the medial surface of the mesonephroi (Figure 7A). Growth of the ovaries is evident throughout development. The diameter of these gonads is presented in Figure 6C and Supplementary Table S6. In contrast to the testes, the cortex of the developing ovaries increases in thickness (Figure 6C; Supplementary Table S7). The mean number of germ cells visible in the cortex per transverse section also increased markedly and is presented in Figure 6D and Supplementary Table S8.

The diameter of the ovarian medulla increased markedly (Figure 6C; Supplementary Table S9). However, the mean number of germ cells visible in the medulla per single ovarian section did not change significantly (Figure 6D; Supplementary Table S10).

**Figure 8:**
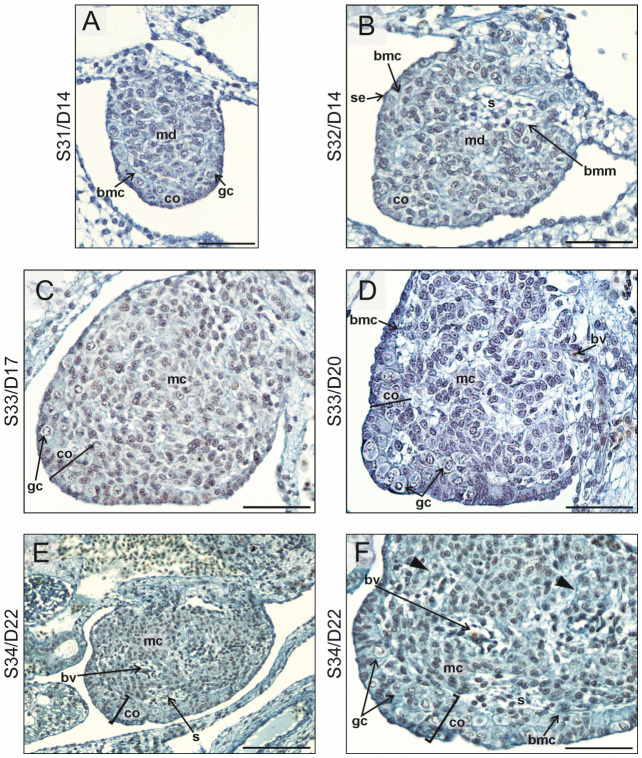
Developing ovaries at subsequent stages: S31/D14 (**A**), S32/D14 (**B**), S33/D17 (**C**), S33/D20 (**D**), and S34/D22 (**E**,**F**). At these stages, a progressive enlargement of the medulla (md) is evident. The cortex (co) forms the peripheral region of the ovary and contains scattered germ cells (gc). In S32/D14 ovaries (**B**), the cortex is covered by a superficial epithelium (se) and is separated from the medulla by a cortical basement membrane (bmc). Medullary cords (mc) are surrounded by basement membranes (bmm), while the intervening tissue constitutes the stroma (s). At S33/D20 and S34/D22 (**D**–**F**), blood vessels (bv) become apparent within the stroma. In panel F, arrowheads indicate fragments of the basement membranes of the medullary cords. Scale bars: (**A**–**D**,**F**)—50 μm; (**E**)—100 μm.

**Figure 9:**
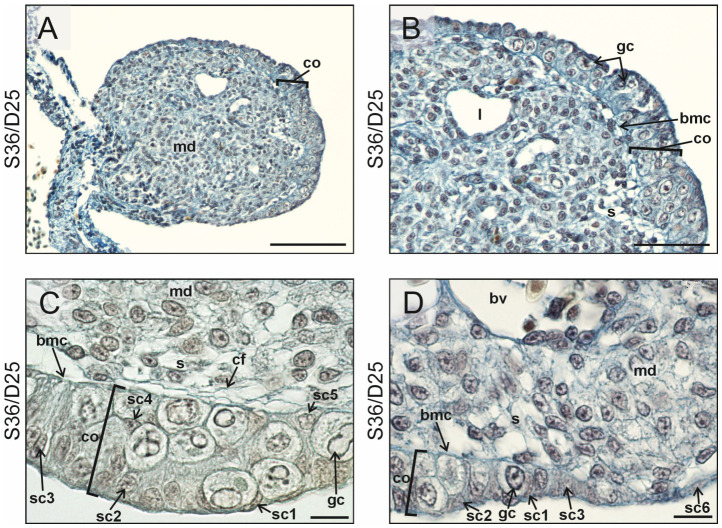
Developing ovaries at S36/D25 (**A**–**D**). A clear subdivision into an extensive medulla (md) and a cortex (co) is visible; the cortex surrounds the gonad and is thickened at the pole opposite to the gonadal attachment. Within the medullary cords, a lumen (l) appears. (**C**) Detail of the thickened cortex. The cortex is separated from the gonadal interior by a basement membrane (bmc) and is composed of somatic cells of varying morphologies (sc1–sc5): sc1—flattened cells directly surrounding the germ cells, with crescent-shaped nuclei; sc2—cells located at the gonadal surface, with spherical nuclei; sc3—elongated cells arranged in a palisade pattern, extending from the gonadal surface to the basement membrane of the cortex, with oval nuclei; sc4—cells located within the cortex, with irregularly shaped nuclei whose shape is determined by the pressure exerted by the surrounding germ cells; sc5—cells located adjacent to the basement membrane of the cortex. Between the medulla (md) and the cortex, stromal cells (s) are dispersed among collagen fibers (cf). Germ cells (gc) are located at the gonadal surface, at the basement membrane of the cortex, and within the cortex interior. (**D**) The lateral surface of the gonad, showing the transition from the thickened cortex to a thin, germ cell-free monolayer of squamous epithelial cells (sc6). Scale bars: (**A**)—100 μm; (**B**)—50 μm; (**C**,**D**)—10 μm.

**Figure 10:**
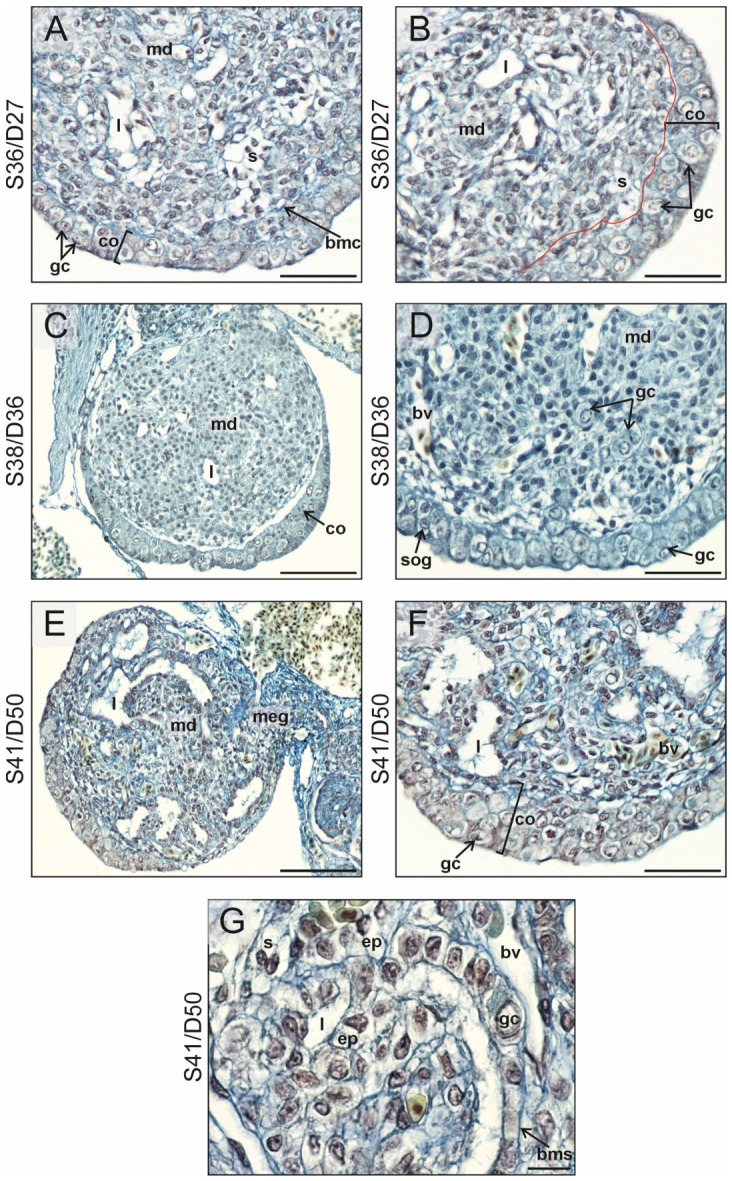
Developing ovaries at S36/D27, S38/D35, and S41/D50. (**A**,**B**) At S36/D27, the ovary consists of an extensive medulla (md) surrounded by a cortex (co). In the ovarian medulla, the medullary cords are transformed into the epithelium lining the luminal spaces (lacunae). The epithelium of the lacunae is separated from one another and from the cortex by the stroma (s). The cortex is separated from the inner part of the gonad by the basement membrane of the cortex (bmc), the course of which is indicated by the red line. Numerous germ cells (gc) are clearly visible within the cortex. (**C**,**D**) In ovaries at S38/D35, the overall organization is similar to that observed at S36. However, blood vessels (bv) within the stroma and persistent germ cells in the medulla are clearly visible in this specimen. In the cortex, some germ cells possess smaller, more darkly stained nuclei, suggesting that they represent secondary oogonia (sog). (**E**–**G**) In ovaries at S41/D50, a distinct accumulation of germ cells is present within the cortex. The cortex is thickest in the region opposite to the mesogonium (meg). The medulla contains large lumina (lacunae). Higher magnification (**G**) shows that these lacunae are lined by a simple epithelium (ep) derived from the medullary cords, among whose cells single germ cells are present. Scale bars: (**A**,**B**,**D**,**F**)—50 μm; (**C**,**E**)—100 μm; (**G**)—10 μm.

In all examined stages, a division into cortex and medulla is visible in the developing ovaries (Figure 7, Figure 8, Figure 9 and Figure 10). The medulla is formed by medullary cords which, in contrast to the testis cords in developing male gonads, are not clearly separated from one another (e.g., Figure 7B–D). The mean diameter of medullary cell nuclei is 5.4 ± 0.3 μm. Unlike the testis cords, the cords of the ovarian medulla at early developmental stages do not show clear epithelial differentiation (compare Figure 4C and Figure 8D). Single germ cells are present among the somatic cells of the medullary cords within the ovarian medulla across all examined stages (e.g., Figure 7B). Extensions of the medullary cords reaching the nearest renal corpuscles of the mesonephroi run through the gonadal mesentery of both sexes and represent the primordium of the excurrent ducts (Figure 7E).

In the ovaries, stroma is visible between the medullary cords, representing the differentiating connective tissue of the ovary that separates the cords, which resemble irregularly distributed cell clusters (Figure 7C). From stage S33/D20 onward, the amount of stroma is substantial (Figure 8D and subsequent figures). It takes the form of dispersed cells suspended in extracellular matrix (Figure 9C). At stage S33/D20, blood vessels are visible at the border between the gonad and mesonephros; however, by S34/D22 they are dispersed within the stroma inside the ovary (Figure 8E,F).

At stage S36, a lumen appears within the medullary cords in developing ovaries (Figure 9B,D). Initially, it takes the form of small, irregular spaces, which correspond to the lacunae described in the medullary region of developing gonads in other reptiles and birds. By stage S41, the center of the ovary is filled with large, irregular spaces (lacunae) (Figure 10E–G). These spaces are lined by a simple cuboidal epithelium, locally transitioning to squamous epithelium (Figure 10G). The basement membranes that previously surrounded the medullary cords are retained and now underlie the epithelial lining of the lacunae. This arrangement results from the transformation of medullary cord cells into the epithelial cells lining the lacunar spaces. Occasional germ cells are incorporated within this epithelium, representing remnants of the germ cells that were previously enclosed within the medullary cords (Figure 10G).

The cortex is the region of the ovary that undergoes less pronounced changes during the examined developmental stages. The thickness of the cortex covering the ovary varies within a single transverse section. The thickest cortex covers the surface of the ovary located furthest from its mesentery, giving the cortex a crescent-shaped appearance in transverse section (Figure 8E, Figure 9A and Figure 10C). Germ cells are most numerous in the region of the thickest cortex (Figure 9C). Near the junction of the ovarian surface with the mesentery, the cortex takes the form of a simple cuboidal epithelium, and in later stages a simple squamous epithelium (Figure 9D). The somatic cells here are differentiated and six cell types can be distinguished: sc1—flattened cells directly surrounding the germ cells, with crescent-shaped nuclei; sc2—cells located at the gonadal surface, with spherical nuclei; sc3—elongated cells arranged in a palisade pattern, extending from the gonadal surface to the basement membrane of the cortex, with oval nuclei; sc4—cells located within the cortex, with irregularly shaped nuclei whose shape is determined by the pressure exerted by the surrounding germ cells; sc5—cells located adjacent to the basement membrane of the cortex; sc6—flattened cells of the simple squamous epithelium covering the gonadal surface near the gonadal mesentery, in the region lacking germ cells.

In the early stages of ovarian development (S29/30–S33), cortical germ cells are irregularly dispersed among numerous somatic cells and do not form distinct layers (Figure 7B and Figure 8D). In later stages, however, as the number of germ cells increases, the cortex thickens and the germ cells form at least two layers (Figure 8F, Figure 9B and Figure 10D). The majority of observed germ cells were at the oogonial stage. They share similar features with germ cells of undifferentiated gonads and spermatogonia. Their nuclei are spherical and lightly stained, with 1–2 visible nucleoli, and the chromatin forms clusters at the nuclear periphery. At stage S38, some cortical germ cells of the ovaries are smaller and have smaller, more darkly stained nuclei (Figure 10D). These may represent secondary oogonia. No meiotic cells were observed in any of the examined stages.

## 4. Discussion

**Figure 11:**
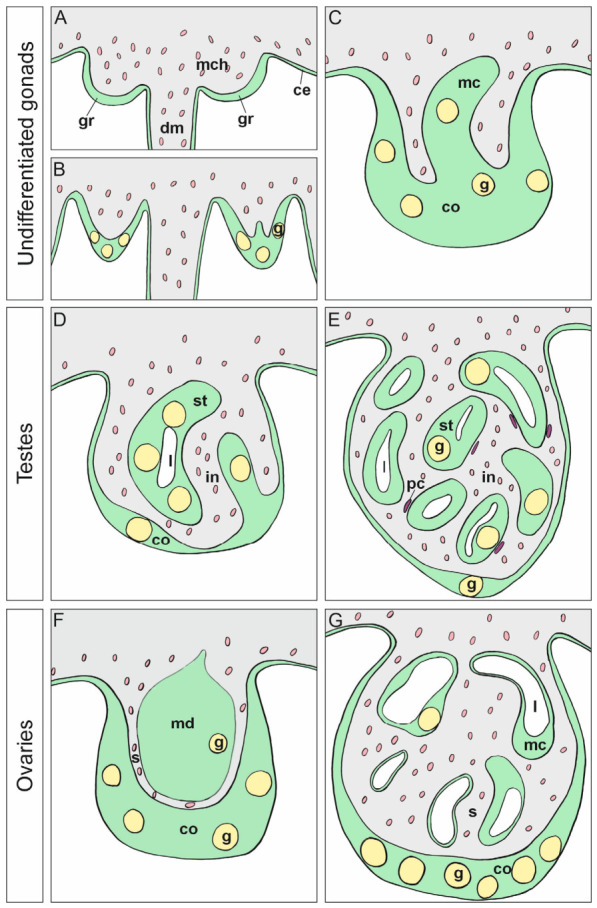
Schematic representation of structural changes during gonadal development in *P. vitticeps*. (**A**) Gonadal ridges (gr) begin to form as two thickenings of the coelomic epithelium (ce) on both sides of the dorsal mesentery (dm); mesenchymal cells (mch) are scattered near the coelomic epithelium and occupy the central region of the gonadal primordia. (**B**) Gonadal ridges are then colonized by germ cells (g). (**C**) The undifferentiated gonads protrude into the body cavity; their surface is formed by a thickened epithelium that constitutes the primitive cortex (co), from which medullary cords (mc) extend into the central region of the gonads. (**D**) Differentiating testes are recognizable by the presence of well-defined testis cords within the gonadal medulla. These cords contain germ cells and rapidly develop a central lumen (l), marking their early transformation into seminiferous tubules (st). (**E**) As testicular development proceeds, seminiferous tubules become more numerous; some interstitial cells (in) surrounding the seminiferous tubules differentiate into peritubular cells (pc); the testes are covered by a thin epithelium with only a few germ cells remaining locally. (**F**) In differentiating ovaries, germ cells are retained in the cortex, while the medulla (md) forms in the center of the gonad, consisting of poorly defined medullary cords; a stroma (s) separates the cortex from the medulla. (**G**) At later stages of ovarian development, germ cells are abundant in the cortex, and a lumen (lacunae) appears within irregularly arranged medullary cords.

The findings of this study illustrate a sequence of morphological transitions initiated at the gonadal ridges and progressing through the undifferentiated gonads (the sex determination period), ultimately leading to the formation of ovarian and testicular structures. These developmental transitions are schematically summarized in Figure 11.

### 4.1. Formation of Gonadal Ridges and Colonization by Primordial Germ Cells

In the bearded dragon (*P. vitticeps*), the earliest morphological signs of gonadal ridge formation were observed at stage S28 (embryonic day 8), when the coelomic epithelium between the dorsal mesentery and the mesonephros begins to thicken bilaterally. This timing is broadly consistent with observations in other squamates. The formation of bipotential gonads in *P. vitticeps* was previously described at a comparable developmental stage (stage 4 according to Whiteley and colleagues [27]) [18]. In geckos (*E. macularius*, *C. ciliatus*) and the veiled chameleon (*C. calyptratus*), gonadal ridge formation likewise precedes PGC colonization [7,20].

The PGCs in *P. vitticeps* are readily identifiable by their characteristic morphology: large, round cells with lightly stained, spherical nuclei consistent with PGC morphology reported in other reptiles [5,17]. The thickened coelomic epithelium forms the primordium of the gonadal cortex and shows evidence of early sex cord formation as epithelial protrusions into the underlying mesenchyme, a feature also described in other squamates [5,7,12,17,20].

### 4.2. The Undifferentiated Gonad Stage

By stage S28/29, the gonadal ridges in *P. vitticeps* have already acquired the structure of undifferentiated gonads, with a recognizable cortex and medulla. The medullary sex cords connect proximally to the cortex and distally to the wall of the renal corpuscles, indicating a structural and potentially functional link to the mesonephros—a feature described across vertebrates and representing the primordium of the excurrent duct system [5,8]. The use of semi-thin sections in the present study enabled the visualization of medullary cord formation—including testis cords in male gonads—arising from the primordial cortex of the gonad, a process already discernible at the stage of undifferentiated gonads. The proximal end of the cords was consistently found to be anchored in and continuous with the surface epithelium. An analogous organization was observed in geckos, chameleons, turtles, and alligators [5,7,12,20]. Collectively, these findings point to a pivotal role of the proliferative activity of the gonadal surface epithelium in driving sex cord development. Germ cells are present in both the cortex and the medullary cords at this stage, which is characteristic of the undifferentiated gonads in reptiles generally [7,10,12,20]. This is consistent with earlier histological observations in *P. vitticeps* [18], who described bipotential gonads at stages 4–8 as elongated structures loosely attached to the mesonephros.

The undifferentiated gonad stage in *P. vitticeps* is notably brief. The transition from the earliest gonadal ridge to a morphologically distinguishable undifferentiated gonad occurs within a single developmental stage, and sex differentiation is already apparent by S29/30. This may be related to differences in the duration of embryonic development, the mode of sex determination, or species-specific developmental rate. The period of undifferentiated gonads represents the developmental window during which sex-determining genes are expressed and temperature may exert its influence on sex determination. Consequently, the precise characterization of this period in *P. vitticeps* is of critical importance for future investigations into the mechanisms of sex determination in this species.

### 4.3. Onset of Sexual Differentiation of Gonads

The first morphological differences between male and female gonads in *P. vitticeps* were observed at stage S29/30 (embryonic day 9). This early onset of sexual differentiation—occurring only one day after the gonadal ridges can first be recognized—is remarkable compared to other species. In other reptiles, the period of undifferentiated gonads is longer, and sexual differentiation of the gonads occurs after eight days of the undifferentiated gonad period in the corn snake; it also takes several days in geckos, turtles, and the American alligator [5,7,9,12,20].

The structure of differentiating testes and ovaries in *P. vitticeps* has previously been described at stages 8 and 9 of the Whiteley staging system [18,27], which correspond to Wise stages 34 and 35 [23] staging system (Supplementary Table S2). Based on the findings of the present study, however, these stages cannot be regarded as the onset of gonadal differentiation, but rather represent a developmental period in which the sex-specific gonadal architecture is already established. The earliest manifestations of sexual differentiation of the gonads can only be identified through rigorous histological analysis.

At S29/30, the distinguishing features of differentiating testes include the pronounced development of testis cords with clear epithelial differentiation and the formation of a distinct basement membrane. The rapid development of a lumen within the testis cords lead to their early transformation into seminiferous tubules, which are already evident in differentiating testes. Simultaneously, the cortex becomes thin and retains only scattered germ cells. In differentiating ovaries at the same stage, the cortex remains thick and multi-layered with a higher number of germ cells, while the medullary cords are loose, irregular and lack a lumen and show no clear epithelial differentiation. This pattern—early thinning of the cortex in testes and retention of a thick cortex in ovaries—is consistent with the general vertebrate model of gonadal sex differentiation [5,9,10,12] and has been documented in other squamates [7,17,18,20]. It also provides the morphological baseline against which the effects of temperature-induced sex reversal can be assessed.

### 4.4. Testis Development

Throughout the examined developmental stages, the testes of *P. vitticeps* show a consistent organizational pattern: an interior filled with seminiferous tubules containing germ cells and a central lumen, surrounded by interstitium, and covered by a thin surface epithelium. The diameter of seminiferous tubules remains constant across stages, suggesting that tubule growth occurs primarily through elongation and coiling. A similar pattern of seminiferous tubules elongation and coiling during embryonic development has been described in other lizards [7,16,20] and in mammals [28].

A particularly notable finding is the apparent change in germ cell proliferative activity during testicular development. Germ cells initially increase in number during gonadal ridge formation and the undifferentiated gonad stage, indicating active proliferation prior to sexual differentiation. In contrast, an apparent mitotic arrest of germ cells within the testis cords is observed during stages S29/30–S33. The number of germ cells per transverse section remains low and relatively constant across these stages, consistent with the entry of prospermatogonia into mitotic arrest—a phenomenon well documented in mammals [29]. The marked increase in germ cell number observed at S36 indicates the resumption of mitotic activity, likely corresponding to the onset of active spermatogonial proliferation. This pattern suggests that the regulatory mechanisms governing prospermatogonial quiescence may be conserved across amniotes.

The Sertoli cells within the seminiferous tubules exhibit a basal position, oval nuclei, and at S36 become more regularly arranged with wider spacing between neighboring cells—consistent with the maturation of the Sertoli cell population. The appearance of interstitial cells, blood vessels, and putative peritubular cells at S36 marks the establishment of a more differentiated testicular architecture, comparable to late embryonic stages described in other squamates [17].

### 4.5. Ovarian Development

Early studies assumed that testes develop from the medulla and ovaries from the cortex [4]; however, it has become evident that during ovarian development the medulla undergoes substantial growth and constitutes the largest portion of the ovary by volume. Our observations of medullary expansion during ovarian development are at odds with the previously reported reduction in medullary size in the developing ovaries of *P. vitticeps* [18]. The number of germ cells in the ovarian medulla remains relatively stable, while the medullary diameter increases substantially—driven by the expansion of cords, stroma and cavity within the medullary cords. The presence of a developing ovarian cavity in *P. vitticeps* indicates that the medullary cords undergo epithelial differentiation and transform to the simple cuboidal-to-squamous epithelium lining the luminal spaces. Formation of cavities has also been described in the ovaries of fish, amphibians, other reptiles, and birds [30,31]. Although in fish and amphibians they reach large sizes, in the ovaries of birds and reptiles they form numerous small, irregular spaces (lacunae). We have previously described small, irregular spaces in the ovarian cortex of geckos and the veiled chameleon [7,20]. Similarly, small spaces have been reported in the ovaries of turtles and alligators [32,33,34]. The spacious lacunae observed in *P. vitticeps* may represent a species-specific feature of ovarian architecture. However, given that large clutch sizes also occur in species with much smaller ovarian cavities, such as *C. calyptratus*, the functional significance of these structures remains unclear.

In contrast to the medulla, the ovarian cortex does not exhibit a marked increase in volume. It is thickened at the pole of the gonad opposite to the mesovarium, where the germinal cells are located. The overall architecture of the embryonic ovary in *P. vitticeps*—characterized by a relatively uniform cortex surrounding the large medulla—closely resembles that previously described in the veiled chameleon, *C. calyptratus* [20]. This structural similarity likely reflects the phylogenetic relatedness of the two species, as Agamidae and Chamaeleonidae are sister families forming the clade Acrodonta. The cortical germ cells in *P. vitticeps* remain at the oogonial stage throughout the examined developmental period (S29/30–S41), with no meiotic cells observed in any individual. At the same time, the continuous increase in germ cell number within the ovarian cortex indicates ongoing oogonial proliferation throughout this period. The onset of meiosis in this species occurs after S41, i.e., beyond embryonic day ~50 under the incubation conditions used in this study. The timing of meiosis initiation varies considerably among reptiles. In the slider *Trachemys scripta* and the European pond turtle Emys orbicularis, meiosis begins during embryogenesis, whereas in the sea turtle *Lepidochelys olivacea*, meiotic cells appear months after hatching [9,35,36]. The timing of meiosis initiation also varies among squamates. The absence of meiotic figures in the embryonic ovaries of *P. vitticeps* is consistent with the pattern described in geckos and the veiled chameleon [7,20], whereas meiotic cells have been identified in the embryonic ovaries of the corn snake [37]. The molecular mechanisms underlying the timing of meiosis initiation remain to be elucidated.

### 4.6. P. vitticeps as a Potential Model Organism for Gonadogenesis Research

The results of the present study demonstrate that *P. vitticeps* offers a detailed and tractable system for studying the morphological events of gonadogenesis in squamates. Several practical advantages support its potential as a model organism, e.g., eggs are readily available from private breeders, females deposit up to 30 eggs per clutch and may produce as many as five clutches per breeding season, and therefore large numbers of biological samples can be obtained from a single pair. These logistical advantages are comparable to those offered by established reptilian models such as *T. scripta* or *Alligator mississippiensis*, but with the additional benefit of a shorter incubation period and a well-characterized sex determination system.

The sex determination system of *P. vitticeps* is of particular interest. This species employs a ZZ/ZW chromosomal system with female heterogamety; however, temperature exerts a significant overriding influence: eggs incubated above 32 °C produce fertile females from ZZ (genetically male) individuals [22]. The comprehensive histological characterization of normal gonadal development provided in the present study is an indispensable prerequisite for any experimental investigation of sex reversal. This point is underscored by a study that used gonad histology as the primary tool to characterize gonadal phenotypes in temperature-switching experiments, identifying not only ovaries and testes but also ovotestes—gonads with mixed male and female morphology that arise as an intermediate phenotype during sex reversal [38]. The morphological baseline established in the present study will facilitate the identification of subtle departures from normal development in future experimental studies using sex-reversing incubation conditions. The precise mechanisms by which temperature influences the specific cellular processes leading to testis or ovary development remain poorly understood and need further investigation. Such studies would complement existing molecular analyses and contribute to a broader understanding of the plasticity of gonadal differentiation in vertebrates.

## 5. Conclusions and Future Directions

The present study provides the first comprehensive morphological description of gonadogenesis in *P. vitticeps*, from the formation of gonadal ridges through the establishment of definitive testicular and ovarian architecture. The data reveal that gonadal sex differentiation in this species begins early (S29/30), that testicular development is characterized by cord/tubule elongation and transient germ cell quiescence, and that ovarian development involves oogonial proliferation in the cortex, medullary expansion, and the formation of lacunae in the medulla. These findings provide an essential morphological framework for future molecular, genetic, and experimental studies of sex determination and gonadal differentiation in this species and in squamates more broadly. Further investigations should aim to elucidate the influence of sex-determining genes and incubation temperature on key cellular processes, including germ cell survival within the ovarian cortex and within the testicular medullary cords, as well as the rapid differentiation of epithelial cells giving rise to the solid testis cords.

## Data Availability

The raw data supporting the conclusions of this article will be made available by the authors on request.

## Supplementary Materials

The following supporting information can be downloaded at: https://www.mdpi.com/article/10.3390/biology15120977/s1, Table S1. Number of bearded dragon (*P. vitticeps*) embryos examined. Table S2. Developmental stages. Table S3. Testis diameter in cross-section. Table S4. Number of testis cords/seminiferous tubules visible per cross-section of testes. Table S5. Number of germ cells per cross-section of testes. Table S6. Ovarian diameter in cross-section. Table S7. Thickness of the ovarian cortex in cross-section. Table S8. Number of germ cells in the ovarian cortex per cross-section. Table S9. Diameter of the ovarian medulla in cross-section. Table S10. Number of germ cells in the ovarian medulla per cross-section.
