# Supplementary material for: Gonadogenesis in the Bearded Dragon (Pogona vitticeps, Agamidae): A Comprehensive Histological Analysis from Gonadal Ridge Formation to Testicular and Ovarian Development
Source: Biology (Basel). 2026 Jun 22;15(12):977. doi: 10.3390/biology15120977 (PMC13297084; doi:10.3390/biology15120977)
Supplement: Supplementary file 1 [file biology-15-00977-s001.zip › biology-4365700-supplementary.pdf]

Supplementary Table S1. Number of bearded dragon (*P. vitticeps*) embryos examined.

Day (days of incubation), Stage (developmental stage according to Wise et al. 2008), Undifferentiated (number of embryos with gonadal ridges and undifferentiated gonads), Males (number of embryos with testes), Females (number of embryos with ovaries), Sum (sum of embryos examined).

Day	Stage	Undifferentiated	Males	Females	Sum
8	28	4	0	0	4
8	28/29	2	0	0	2
9	29	4	0	0	4
9	29/30	0	4	3	7
11	30	0	3	4	7
13	30	0	0	1	1
11	31	0	3	3	6
12	31	0	2	3	5
14	31	0	1	1	2
14	32	0	3	2	5
15	33	0	2	3	5
17	33	0	2	0	2
20	33	0	1	1	2
22	34	0	0	1	1
24	34	0	0	1	1
25	36	0	4	2	6
27	36	0	0	3	3
36	38	0	0	2	2
50	41	0	0	1	1

Supplementary Table S2. Correspondence between developmental stages defined by Wise et al. (2009) and Whiteley et al. (2017), with the diagnostic characteristics used for stage identification.

Stage according to Wise et al. (2009)	Stage according to Whiteley et al. (2017)	Diagnostic features
28	1	Forelimb buds are visible as small protrusions, while hindlimb buds are not yet visible.
28/29	1/2	The first signs of hindlimb buds are visible as small protrusions.
29	2	Forelimb and hindlimb buds are distinct, with the forelimb buds being larger.
29/30	3	Forelimb buds become plate-like.
30	5	The limb buds are plate-shaped with distinct, round margins.
31	6	Forelimbs and hindlimbs are similar in appearance, and the autopodium is flat, paddle-shaped, and roughly triangular.
32	7	The forelimbs and hindlimbs are differentiated into stylopodium, zeugopodium, and autopodium, with no visible digital condensations in the autopodium.
33	8	Condensations of digits 2–4 are visible in the autopodia of both the forelimbs and hindlimbs.
34	8	Condensations demarcating all five digits are visible in both the forelimb and hindlimb autopodia.
35	9	The interdigital webbing is deeply incised, with digits 1 and 5 noticeably shorter than digits 2–4.
36	10	Interdigital webbing is absent, and phalangeal segments are beginning to form within the digits.
38	13	Scales begin to develop on the forelimbs during this stage and completely cover them by its end.
41		The external nares are present as distinct pits and remain closed.

Supplementary Table S3. Testis diameter in cross-section.

Stage	Individuals	Mean testis diameter [μm]	SD [μm]
29/30	n=4	113.8	4.3
30	n=3	129.4	8.3
31	n=6	148.7	7.3
32	n=3	153.5	12.3
33	n=5	168.4	13.3
36	n=4	239.6	17.3

All values represent means of measurements performed in the widest central region of the gonads.

Supplementary Table S4. Number of testis cords/seminiferous tubules visible per cross-section of testes.

Stage	Individuals	Mean number of testis cords/seminiferous tubules	SD
29/30	n=4	1.1	0.6
30	n=3	1.6	0.8
31	n=6	2.5	0.8
32	n=3	2.6	0.8
33	n=5	2.9	0.9
36	n=4	9.2	1.1

Supplementary Table S5. Number of germ cells per cross-section of testes.

Stage	Individuals	Mean number of germ cells	SD
29/30	n=4	3.7	0.5
30	n=3	3.8	0.4
31	n=6	3.8	0.6
32	n=3	3.9	0.6
33	n=5	4.2	0.6
36	n=4	11.8	1.5

Supplementary Table S6. Ovarian diameter in cross-section.

Stage	Individuals	Mean ovarian diameter [μm]	SD [μm]
29/30	n=3	112.3	7.3
30	n=5	116.2	8.2
31	n=7	123.4	6.3
32	n=2	142.2	10.2
33	n=4	189.3	11.2
36	n=5	255.3	18.3
38	n=2	331.5	21.3
41	n=1	332.9	19.4

**For S41, only one individual was available; mean and SD were calculated from multiple analysed cross-sections.*

Supplementary Table S7. Thickness of the ovarian cortex in cross-section.

Stage	Individuals	Mean cortex thickness [μm]	SD [μm]
29/30	n=3	17.6	6.3
30	n=5	32.1	6.7
31	n=7	38.3	7.3
32	n=2	40.2	8.4
33	n=4	40.9	9.1
36	n=5	44.3	8.4
38	n=2	46.6	10.3
41	n=1	46.9	12.1

**For S41, only one individual was available; mean and SD were calculated from multiple analysed cross-sections.*

Supplementary Table S8. Number of germ cells in the ovarian cortex per cross-section.

Stage	Individuals	Mean number of germ cells	SD
29/30	n=3	7.4	0.7
30	n=5	8.3	1.5
31	n=7	9.2	1.4
32	n=2	14.6	1.9
33	n=4	16.2	1.6
36	n=5	34.6	4.3
38	n=2	54.2	5.2
41	n=1	56.2	8.4

**For S41, only one individual was available; mean and SD were calculated from multiple analysed cross-sections.*

Supplementary Table S9. Diameter of the ovarian medulla in cross-section.

Stage	Individuals	Mean medulla diameter [μm]	SD [μm]
29/30	n=3	80.4	9.4
30	n=5	81	10.2
31	n=7	83.1	12.2
32	n=2	98.6	14.3
33	n=4	139.5	17.7
36	n=5	201.2	22.5
38	n=2	279.2	28.2
41	n=1	281.3	21.7

**For S41, only one individual was available; mean and SD were calculated from multiple analysed cross-sections.*

Supplementary Table S10. Number of germ cells in the ovarian medulla per cross-section.

Stage	Individuals	Mean number of germ cells in the medulla	SD
29/30	n=3	4.1	1.6
30	n=5	4.5	2
31	n=7	5.1	1.3
32	n=2	4.7	1.9
33	n=4	5.1	1.5
36	n=5	6.5	2.5
38	n=2	5.4	2.1
41	n=1	5.2	1.9

**For S41, only one individual was available; mean and SD were calculated from multiple analysed cross-sections.*
